# Combining Tibial Cortex Transverse Transport (TTT) and Endovascular Therapy (EVT) for Limb Salvage in Chronic Limb‐Threatening Ischemia

**DOI:** 10.1111/os.14222

**Published:** 2024-09-07

**Authors:** Yi Ding, Dapeng Yu, Haoheng Huang, Xiao Peng, Shenghui Yang, Zhanming Lin, Peiling Zhou, Jilin Liang, Xiaochong Zou, Ruiqing Mo, Kaixiang Pan, Puxiang Zheng, Xiaocong Kuang, Xinyu Nie, Qikai Hua

**Affiliations:** ^1^ Department of Bone and Joint Surgery The First Affiliated Hospital of Guangxi Medical University Nanning China; ^2^ Guangxi Diabetic Foot Salvage Engineering Research Center Nanning China; ^3^ Department of Traumatic Orthopedics Affiliated Hospital of Guangdong Medical University Zhanjiang China; ^4^ National Demonstration Center for Experimental General Medicine Education, Xianning Medical College Hubei University of Science and Technology Xianning China; ^5^ Yulin Campus of Guangxi Medical University Yulin China

**Keywords:** Chronic Limb‐Threatening Ischemia, Endovascular Therapy, Limb Salvage, Tibial Cortex Transverse Transport

## Abstract

**Objective:**

The clinical management of patients with chronic limb‐threatening ischemia (CLTI) faces great challenges. Enhancing wound healing and limb preservation rates in this cohort is a critical objective. This study investigates the effectiveness of combining tibial cortex transverse transport (TTT) and endovascular therapy (EVT) for the treatment of patients with severe CLTI. We aim to evaluate the therapeutic results of this combined approach on the specified patient group.

**Methods:**

We conducted a retrospective study to compare EVT with the combination of TTT and EVT in patients (Rutherford category 5 and above) with CLTI at Guangxi Medical University's First Affiliated Hospital from June 2017 to June 2023. This cohort was subjected to a follow‐up period ranging from a minimum of 6 months to a maximum of 12 months. The primary outcome measures included amputation‐free survival (AFS) (avoidance of above‐ankle amputation or death from any cause), overall mortality, limb salvage rates, wound healing efficiency, and the technical efficacy of the applied treatments. A variety of statistical analyses including chi‐square tests, Fisher's exact tests, and Pearson's and Spearman's correlation analyses.

**Results:**

In this study, 131 patients with CLTI were included: 76 in the control group receiving only EVT treatment and 55 in the TTT + EVT group. The two groups were matched on demographic and clinical characteristics. In the TTT + EVT group, after more than 6 months of follow‐up, 85.5% of patients achieved AFS, and wound healing was observed in 54.5% (30 of 55 patients). After more than 12 months of follow‐up, 81.9% achieved AFS, with wound healing in 32 patients. Furthermore, after more than 24 months, 74.2% of patients remained amputation‐free, with wound healing in all surviving patients. In the control group, after more than 6 months of follow‐up, 72.4% of patients achieved AFS, and wound healing was observed in 51.3% (39 of 96 patients). After more than 12 months, 48.9% achieved AFS, with wound healing in 21 patients.

**Conclusion:**

We found that combining therapy of TTT and EVT is safe and can be successfully administered in patients with CLTI and it enhances wound healing and AFS.

## Graphical abstract

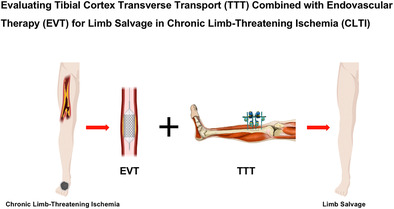

## Introduction

Chronic limb‐threatening ischemia (CLTI) manifests as a complex clinical syndrome, primarily characterized by persistent pain, gangrene, or persistent lower limb ulcers, often linked to peripheral arterial disease. This condition emerges distinctly after ruling out other etiologies such as venous, traumatic, embolic, and non‐atherosclerotic causes.^1^ In the United States, the prevalence and incidence of CLTI among individuals over 40 years are approximately 1.33% and 0.35%, respectively. This translates to nearly 1 million affected individuals within the Medicare demographic alone.^2^, ^3^, ^4^ Without effective therapeutic interventions, most CLTI cases—characterized by persistent pain, nonhealing wounds, and gangrene—progress to major amputations above the ankle. This is particularly alarming in elderly patients (over 65 years), where major amputations are associated with a 50% mortality rate within the first year, a rate that increases further in those with cardiovascular diseases.^2^, ^5^

Currently, endovascular therapy (EVT) such as arterial revascularization stands as the cornerstone treatment modality for CLTI, particularly in its most advanced stages, as seen in CLTI patients.^6^, ^7^ These patients are predominantly at an escalated risk of amputation within a year. Effective vascular reconstruction techniques are pivotal in the limb salvage therapeutic strategy for CLTI patients. Despite the technical success of EVT in averting amputation in certain cases, patients with CLTI continue to confront a heightened risk of limb loss and mortality. This scenario underscores the urgent need for innovative technological advancements to enhance patient outcomes in CLTI.^8^, ^9^

Tibial Cortex Transverse Transport (TTT), a novel technique derived from the Ilizarov method, offers a promising approach by facilitating the reconstruction of lower limb blood flow. Our preliminary investigations have applied TTT in the management of severe diabetic foot ulcers compounded by peripheral vascular disease, with encouraging outcomes indicating enhanced foot perfusion and improved wound healing rates.^10^, ^11^, ^12^ This study, therefore, aims to critically evaluate the therapeutic potential of TTT, in conjunction with EVT for limb salvage in patients with CLTI. The objective is to ascertain the efficacy and applicability of TTT as a valuable addition to the current treatment paradigms for CLTI. The purpose of the study was to evaluate whether the combination of TTT and EVT improves amputation‐free survival (AFS) in patients with severe CLTI and improves the rate of ulcer wound healing in other patients.

## Patients and Methods

### Study Design and Setting

This study is a clinical retrospective investigation, reviewing patients treated from June 2017 to June 2023. Patients with Rutherford classification 5 (tissue loss or focal gangrene) or classification 6 (extensive gangrene) CLTI were eligible for enrollment. They either underwent TTT + EVT or received EVT treatment alone. Data collection was part of routine patient follow‐up examinations. The study was approved by the Ethics Committee of the First Affiliated Hospital of Guangxi Medical University(2023‐E462‐01).

### Patient Inclusion and Exclusion Criteria

During this period, we treated 131 patients, all at least 18 years old, with CLTI classified as Rutherford class 5 or 6. Patients in the TTT group received combined TTT and EVT treatment, while the control group received EVT treatment only. We applied strict exclusion criteria to ensure the integrity of the study outcomes: (1) development of advanced malignancies or other terminal conditions post‐intervention; (2) initiation of treatment with cytotoxic agents, corticosteroids, or other immunosuppressive therapies, as well as exposure to radiotherapy and chemotherapy posttreatment; (3) inadequate follow‐up period, defined as less than 6 months.

### Surgical Technique

#### TTT

**FIGURE 1:**
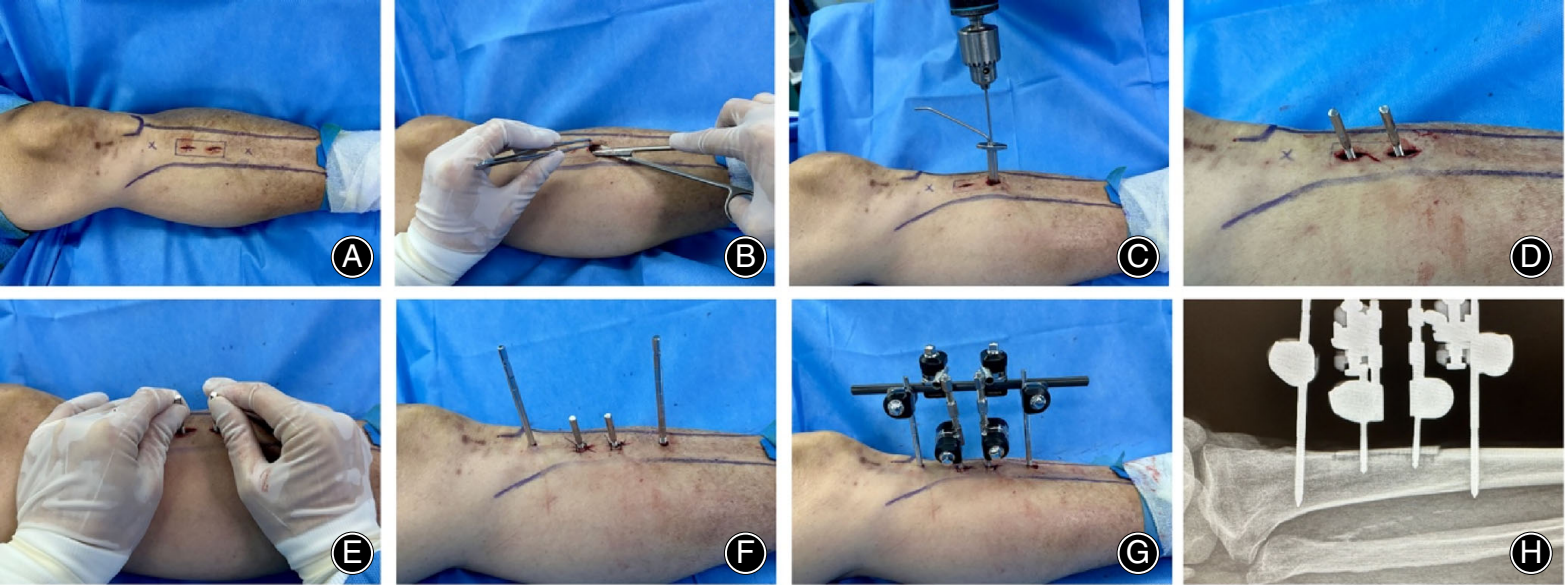
Surgical procedure for Tibial cortex transverse transport (TTT). (A) Preoperative marking of the surgical area and creation of two longitudinal skin incisions. (B) Dissection and exposure of the subcutaneous tissue over the periosteal surface. (C) Execution of successive perforations to facilitate pre‐truncation. (D) Insertion and securing of the transfer pin. (E) Isolation and removal of the bone block. (F, G) Insertion of fixation pins and assembly of the external fixation and transfer apparatus. (H) Postoperative radiographic confirmation of successful corticotomy interception.

Mark a rectangular area of approximately 5 cm in length and 1.5 cm in width on the medial aspect of the tibial diaphysis. Proceed to create two longitudinal incisions, each about 1‐cm long, within the confines of this demarcation, carefully incising only the skin layer. Diligently preserve the periosteum and meticulously avoid disrupting the adjacent vasculature and neural structures. Employ a fine osteotome to methodically perform a corticotomy within the predefined area, maintaining the dimensions of the cortical window. Fixate a 3‐mm transfer pin onto the osteotomized bone segment to facilitate its mobilization. Insert 5‐mm Schanz pins transversely at either end of the mobilized segment, ensuring a separation of approximately 3 cm between them, and allow for a 2‐mm protrusion beyond the contralateral cortical surface. Assemble the external fixator's transport mechanism. Postoperatively, obtain radiographic confirmation to ascertain the precision of the pin placement and the integrity of the osteotomy (Figure 1).

#### EVT

Based on preoperative CT angiography (CTA) and lower extremity color Doppler ultrasonography, we identified the lesion locations and selected appropriate puncture sites. Following successful anesthesia, an arterial sheath was inserted at the predetermined puncture site as per the preoperative plan. Intraoperative digital subtraction angiography (DSA) was utilized to further delineate the lesion. Percutaneous transluminal angioplasty (PTA) was the preferred initial treatment. If the balloon expansion was unsatisfactory, indicated by persistent pressure gradient across the lesion, residual stenosis >50%, or the occurrence of flow‐impeding dissection, a vascular stent was then deployed.

### Postoperative Management and Follow‐Up

**FIGURE 2:**
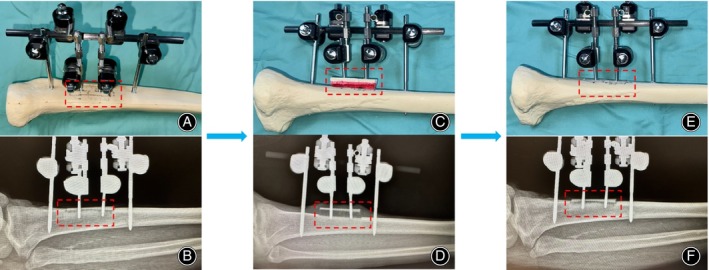
Corticotomy mobilization Post‐T Tibial cortex transverse transport (TTT). (A, C, E) Demonstration using a prosthetic bone to simulate the surgical procedure and the corticotomy adjustment process. (B, D, F) X‐ray images of the patient at varying postoperative stages. (A,B) Directly post‐operation, depicting the osteotomized bone window along with the external fixation mobilization device in place. (C,D) At 10 days post‐adjustment, the corticotomy reaches its apex; subsequent radiographs confirm the precise positioning of the corticotomy and validate the success of the upward adjustment process. (E, F) Ten days following the reversal adjustment of the external fixation device, radiographs are taken to verify the bone block's return to its original position.

Beginning on the fifth postoperative day, adjustments to the external fixation frame are initiated to gradually transport the bone block. This involves an outward transport of 1 mm per day, completed in three stages. After 10 days of transport, an X‐ray examination is conducted to ascertain the position of the bone block. Subsequently, the corticotomy is transported backward at the same rate. Following 10 days, during which the tibial bone window is returned to its original position, another X‐ray examination is performed for further assessment and adjustment of the bone block. These steps may be modified based on the patient's clinical presentation (Figure 2).

In our approach to wound care, we adhered to international guidelines for aggressive debridement.^13^ The majority of these debridement procedures were integrated with wound care management. Our objective was not to achieve complete removal of necrotic tissue in each session but rather to employ multiple, smaller‐scale interventions.

All patients were advised to engage in functional rehabilitation, which included walking with assistive devices while ensuring non‐weight bearing on the affected limb. This precaution is essential as the healing process can be significantly hindered by pressure exerted during walking, contact with the bed surface while lying down, or constriction from tight dressings.

### Outcomes Measures

In this study, we included baseline data such as patients' age, gender, smoking history, alcohol consumption history, ulcer location, ulcer size, ulcer duration, and comorbidities. The primary outcomes of the study were the assessment of AFS at 6 and 12 months, while the secondary outcomes focused on evaluating wound healing progress.

### Statistical Analysis

Standard statistical analysis was conducted using SPSS software version 20.0 (SPSS Inc., Chicago, IL). Continuous variables were summarized by mean ± standard deviation (SD), while categorical variables were summarized by count and percentage and the analysis of categorical variables was performed by the chi‐square test, Fisher's exact chi‐square test, or Pearson's chi squared test.

## Results

### Patient Characteristic

From June 2017 to June 2023, 55 patients were diagnosed with CLTI and treated with a combination of TTT and EVT. The study included 55 patients in the TTT group (TTT + EVT) and 76 patients in the control group (EVT). The baseline characteristics of the patients are summarized in Table 1. All patients' affected limbs were classified as Rutherford Grade 5 (ischemic rest pain) or Grade 6 (ischemic ulcers or gangrene). There were no significant differences between the groups in factors associated with ulcer healing, such as age, sex, alcohol consumption, smoking status, diabetes, hypertension, or cerebrovascular disease (*p* > 0.05). However, there were statistically significant differences in Rutherford classification, ulcer duration, ulcer size, renal failure on dialysis, biochemical criteria，and coronary artery disease, with the TTT group representing a more severely affected cohort.

**TABLE 1:** Characteristics of patients.

Characteristics	TTT (*n* = 55)	Control (*n* = 76)	*t*/*z*/X^2^	*p*
Male	43 (78.2%)	58 (76.3%)	0.063	0.802
BMI (kg/m^2^)	21.2 ± 3.1	21.7 ± 3.4	1.002	0.318
Age (years)	71.8 ± 11.8	73.8 ± 10.0	1.071	0.286
Smoking	32 (58.2%)	45 (59.2%)	0.014	0.906
Drinking	20 (36.4%)	31 (48.0%)	0.263	0.608
Rutherford classification				
5	10 (18.2%)	31 (40.8%)	7.585	0.006
6	45 (81.8%)	45 (59.2%)		
Site of ulcer				
Left	26 (47.3%)	43 (56.6%)	1.109	0.292
Ulcer area (cm^2^)	25 (10.60)	13 (9.35)	−2.693	0.007
Osteomyelitis	49 (89.1%)	58 (76.3%)	3.480	0.062
Duration of ulcers (months)	4 (2.8)	2 (1.4)	−2.767	0.006
Diabetes	25 (45.5%)	23 (30.3%)	3.172	0.075
Renal failure on dialysis	10 (18.2%)	5 (6.0%)	4.237	0.040
Hypertension	32 (54.5%)	55 (72.4%)	12.363	0.090
Coronary artery disease	30 (54.5%)	28 (36.8%)	4.053	0.011
Cerebrovascular disease	30 (54.5%)	35 (46.1%)	0.921	0.337
Biochemical criterion				
WBC (×10^9^/L)	13.8 (10.1, 16.4)	9.9 (7.7, 13.5)	−3.663	<0.001

*Note*: Data are presented as the mean ± SD or *n* (%).

Abbreviation: TTT, Tibial cortex transverse transport.

### Clinical Data and Outcomes

The 55 patients of the TTT group received more than 6 months follow‐up and AFS was noted in 47 (85.5%) patients and wound healed in 30 of 55 patients (54.5%).Notably, 44 patients received more than 12 months of follow‐up, with AFS noted in 36 (81.9%) patients, and wound healing observed in 32 patients (Table 2). Compared with the TTT group, the control group had lower healing rates at 6 and 12 months, with healing in 39 (51.3%) and 21 (46.7%) patients, respectively. Additionally, AFS in the control group was lower at 6 and 12 months, with 53 (72.4%) and 22 (48.9%) patients, respectively, showing significant differences compared with the TTT group at each time point.

**TABLE 2:** Outcomes of combining Tibial cortex transverse distraction and endovascular therapy for chronic limb‐threatening ischemia (CLTI).

Follow‐up	TTT (*n*/%)^a^	Control (*n*/%)^a^	X^2^	*p*
6 months	55	76		
Healed	30 (54.5%)	39 (51.3%)	0.134	0.715
Amputation	4 (7.3%)	9 (11.8%)	0.322	0.388
Death	4 (7.3%)	14 (18.4%)	2.471	0.067
AFS	47 (85.5%)	53 (72.4%)	4.364	0.037
12 months	44	45		
Healed	32 (72.7%)	21 (46.7%)	12.363	0.012
Amputation	4 (9.1%)	4 (8.9%)	0.011	0.973
Death	4 (9.1%)	19 (42.2%)	5.757	<0.001
AFS	36 (81.9%)	22 (48.9%)	17.237	0.001

^a^ Data are presented as the *n* (%).

Abbreviation: TTT, Tibial cortex transverse transport.

In the TTT group, limb amputation was necessary in four patients. Two patients underwent amputation after acute arterial occlusion at 2 and 5 months postoperatively, which resulted in limb necrosis. The other two patients experienced persistent infections and worsening of the wound site post‐surgery, with no significant improvement in clinical symptoms following the surgical intervention, ultimately leading to amputation at 2 and 3 months post‐surgery, respectively (Table 2). In the control group, amputation rates were higher at 6 and 12 months, with 9 (11.8%) and 4 (8.9%) patients, respectively, showing significant differences compared with the TTT group at each time point.

Mortality occurred in nine patients during the study period. Six of these deaths were attributed to severe cardiovascular events, while the remaining three patients succumbed to complications following COVID‐19 infection. Mortality rates in the control group at 6 and 12 months were 14 (18.4%) and 19 (42.2%) patients, respectively, showing significant differences compared with the TTT group at each time point.

## Discussion

TTT as a novel technique to promote microvascular neogenesis in the lower limbs has been applied in various ischemic conditions of the lower extremities.^10^, ^12^, ^14^, ^15^ In this study, we employed a combination of TTT and EVT to treat patients with Rutherford grade 5 or higher and compared the outcomes with patients who received EVT alone. We found that the combined treatment of TTT and EVT significantly improved AFS at both 6‐month and 12‐month follow‐up points, which is crucial for patients with CLTI. Additionally, the healing rate in the combined treatment group was significantly higher compared with the control group. Therefore, for patients with severe CLTI, combined therapy with TTT may be a promising new option to enhance AFS.

### AFS, Amputation and Mortality

**FIGURE 3:**
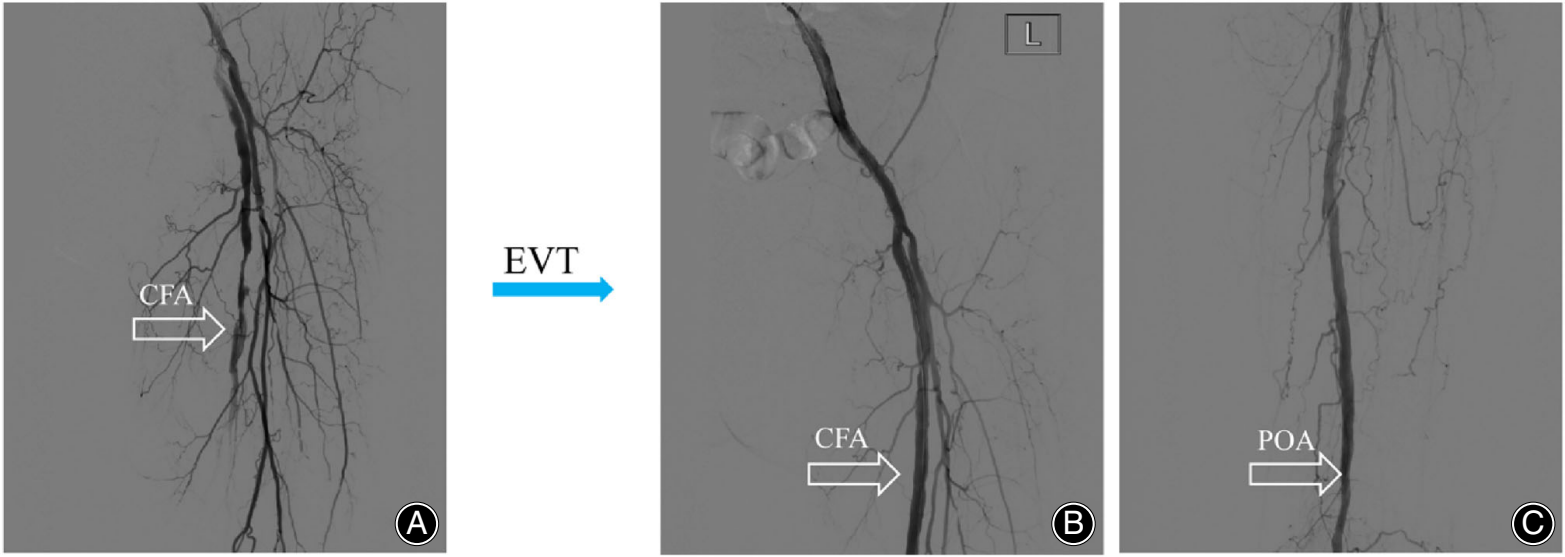
Endovascular intervention (EVT) for a 68‐Year‐Old Patient with Clinical Stage 4 Chronic Limb‐Threatening Ischemia (CLTI). (A) Digital Subtraction Angiography (DSA) during the procedure reveals occlusion of the femoral artery and a complete absence of distal blood flow visualization. (B, C) Post‐EVT imaging demonstrates restored patency in both the femoral artery (CFA) and the popliteal artery (POA), indicating successful recanalization.

**FIGURE 4:**
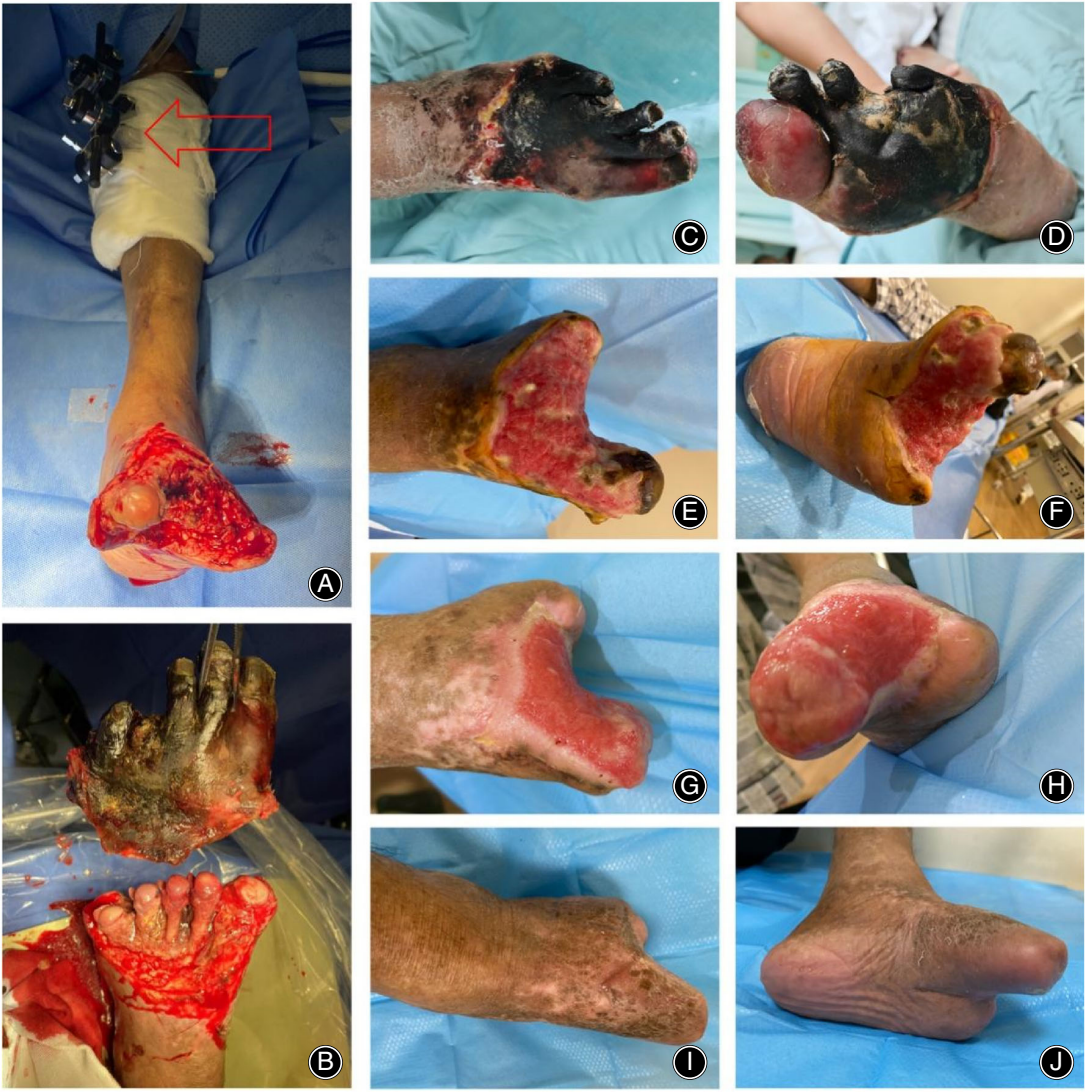
Combined Tibial Cortex Transverse Transport (TTT) and endovascular intervention for trauma management in a patient with chronic Limb‐Threatening Ischemia (CLTI). (A,B) Intraoperative images showing the installation of a transverse tibial bone transfer external frame (indicated by red arrow) and debridement of the trauma. The intraoperative assessment revealed necrosis of the 2nd to 4th metatarsals, which were excised en bloc. The 1st metatarsal was spared as it was enveloped by fascial tissue and exhibited no necrosis. (C, D) Preoperative images displaying dry gangrene of the forefoot. (E, F) Two weeks post‐operation, the trauma site is covered with new granulation tissue; however, secondary necrosis of the exposed metatarsal heads occurred, necessitating bedside removal during subsequent treatments. (G, H) At 6 weeks post‐surgery, images depict healthy granulation tissue covering the trauma, skin growth at the trauma margins, and a reduction in the size of the trauma. (I, J) The final images show the fully healed wound.

In a retrospective analysis involving 849 CLTI patients primarily treated with EVT, a 2‐year mortality rate of 32.3% and an amputation rate of 13.5% were reported in patients at WIFI clinical stage 4.^16^ Another multicenter, open‐label, randomized study of 345 CLTI patients undergoing EVT revealed 1‐ and 2‐year survival rates of 82.1% and 72.3%, respectively, with AFS rates of 73.4% and 64.7%.^17^ Our control group data were also consistent with these reports, showing high rates of amputation and mortality. In contrast, our retrospective study demonstrated superior outcomes in CLTI patients treated with combined EVT and TTT, showing AFS rates of 82.6% and 79.5%, and survival rates of 91.3% and 89.7% at 1 and 2 years post‐surgery, respectively, and at the same time, the patient's CLTI was relieved and the wound healed (Figures 3 and 4).

For amputation, even though TTT can promote wound healing in patients with severely infected diabetic foot, this study still observed two instances where patients developed postoperative infections leading to amputation. This indicates that there remains a need to strengthen postoperative wound care for patients with CLTI.

Regarding mortality, cardiovascular events were a significant cause, but notably, in the TTT group, 33.3% of deaths in our cohort were due to COVID‐19 comorbidities.^18^ Elderly patients with cardiovascular diseases are particularly vulnerable to severe outcomes from COVID‐19, leading to increased mortality and potentially contributing to an underestimation of our survival data.

### Mechanism

While EVT enhances perfusion in large vessels, peripheral perfusion is crucial for the health of tissues such as skin, muscle, fat, and bone, and for successful ulcer healing.^19^ TTT, based on Ilizarov's distraction osteogenesis principles, fosters angiogenesis in diabetic foot patients, improving limb perfusion and ulcer healing.^10^, ^11^, ^12^, ^20^, ^21^ In the animal models developed using TTT technology, it was observed that TTT can promote increased blood flow in the lower extremities and enhance microcirculatory perfusion.^14^, ^22^ This synergistic effect of TTT when combined with EVT likely contributes to the improved rates of limb salvage noted in our observations.

### Perspectives and Limitation

TTT as a novel method for promoting microvascular growth in the lower limbs offers a simpler and less technically demanding alternative compared with traditional EVT or newer endovascular procedures such as transcatheter arterialization of deep veins in CLTI.^23^ Its efficacy has been validated across multiple disease conditions, confirming the therapeutic benefits of TTT. However, our study is subject to several limitations. It was conducted as a single‐center, retrospective study, characterized by a low level of evidence and a limited number of participants. Consequently, our findings necessitate validation through multicenter studies encompassing larger sample sizes. Moreover, the duration of follow‐up in our study was restricted, making it imperative to continue follow‐up assessments for more comprehensive insights.

## Conclusion

Our findings suggest that the combination of TTT and EVT is a safe and effective approach for enhancing limb salvage in patients with CLTI. This combination therapy not only promotes wound healing but also aids in preventing major amputations.

## Conflict of Interest Statement

The authors declare that they have no competing interests.

## Funding Information

This study was supported by grants from the; Guangxi Key Research and Development Plan (2021AB11027); Key Research and Development Plan of Qingxiu District, Nanning City (2020053); National Natural Science Foundation of China (82260448); and Clinical Research Climbing Plan of the First Affiliated Hospital of Guangxi Medical University (YYZS2020010).

## Author Contributions

All authors contributed to the article and approved the submitted version. Yi Ding, Dapeng Yu, and Haoheng Huang performed the data collection, graph production, and manuscript writing. Xiao Peng, Shenghui Yang, and Zhanming Lin performed the data compilation and assist in writing manuscripts. Peiling Zhou, Jilin Liang, Xiaochong Zhou, Ruiqing Mo, Kaixiang Pan, Puxiang Zheng, and Xiaocong Kuang helped perform the analysis through constructive discussions. Xinyu Nie and Qikai Hua contributed to the conception and design of the study.
